# Self-Perception or Objective State: A Further Study of the Effects of Retirement on Health

**DOI:** 10.3389/fpsyg.2022.820972

**Published:** 2022-03-15

**Authors:** Yuanmao Tang, Danping Liu, Shaobo Mou, Salmi Mohd Isa, Siyuan Gui, Qin Wan

**Affiliations:** ^1^Guangzhou Rural Commercial Bank, Guangzhou, China; ^2^School of Management, Xihua University, Chengdu, China; ^3^Research Institute of International Economics and Management, Xihua University, Chengdu, China; ^4^Xihua Honor College, Xihua University, Chengdu, China; ^5^Graduate School of Business, Universiti Sains Malaysia, Penang, Malaysia; ^6^School of Management, Southwest Petroleum University, Chengdu, China

**Keywords:** retirement, subjective health, objective health, lifestyle, fuzzy regression discontinuity design

## Abstract

Against the backdrop of an aging global population and the increasing pressure of medical care expenditures for seniors, this paper used a fuzzy regression discontinuity (FRD) model to explore the effects of retirement on the self-assessed health and objective physical and mental health of older people. Using survey data from the China Health and Retirement Longitudinal Study (CHARLS), our model addresses some relevant academic controversies. Our sample was comprised of male respondents from government agencies, enterprises, and public institutions. The research explored the impact of retirement on lifestyle habits and included an in-depth analysis of the mechanism through which retirement influences different aspects of health. The results show that: (1) Retirement does not have any significant impact on objective health, including depression and self-care ability, but it does cause a notable decline in subjective health assessment. (2) Retirement shortened the sleep time of respondents, which may account for lower scores on subjective health self-evaluations, but it did not lead to any noticeable improvement in habits which are harmful to health, such as smoking and drinking. (3) Marriage can help alleviate the problems of depression and smoking among older people, and education has a somewhat broader positive effect on their health and lifestyles; however, neither factor helps to improve the sleep problems of older people. Therefore, this paper recommends that efforts should be made to both optimize retirement policies and seek further ways to improve the health of the retired population.

## Introduction

Internationally, a country or a region is considered to have an aging society if people over age 60 account for at least 10% of its population or if those over age 65 constitute 7% of the population (Zhang et al., 2021). China is among those countries with a relatively high aged population. In 2020, people over age 60 accounted for 18.7% of the country’s population. As China’s second child-bearing peak population moves into old age, China’s elderly population will begin to grow faster from 2021 to 2050, with an average annual increase of 6.2 million people. This proportion is expected to expand to one-third by 2050. Based on international experience, as a nation’s society progressively ages, problems such as labor shortages and the pressure of social security benefits and medical expenditures for the retired intensify (Grant et al., 2004; Kinsella and Phillips, 2005; Müller and Shaikh, 2018). This is particularly true for emerging economies and developing countries. Some suggest that in order to respond effectively to an aging population, society must make positive changes to retirement policies. According to this argument, raising the retirement age helps eliminate the negative image of older people, making them a resource rather than a burden on society. However, this premise is controversial. Furthermore, from an economic perspective, whether raising the age of retirement can achieve this purpose or simply exacerbates the problem is, to a large extent, determined by the effects of retirement on the health of older people.

According to previous studies, developed countries become aging societies earlier and have thus conducted numerous tracking surveys collecting data about the health of retired and aging populations (Celidoni and Rebba, 2017; Blake and Garrouste, 2019). These include the EU’s Survey of Health, Aging and Retirement in Europe (SHARE), the United Kingdom’s English Longitudinal Study of Aging (ELSA), Japan’s Japanese Study on Aging and Retirement (JSTAR), and the United States’s RAND Health and Retirement Study (HRS). A large body of research literature has been generated based on data from these surveys. However, these studies focus only on developed economies, and no consensus has been reached. Some researchers believe that retirement can improve health outcomes (Coe and Zamarro, 2011; Insler, 2014). According to Mazzonna and Peracchi (2017), retirement reduces one’s workload and increases the time for leisure, rest, and exercise. Kämpfen and Maurer (2016) maintain that the positive influence of retirement on health can be attributed to reduced stress. However, other studies assert that retirement may have a negative effect on the health of the aging population (Behncke, 2012). Dave et al. (2006) posited that this might be related to inadequate income caused by social security funding gaps. Godard (2016) found that retirement may lead to obesity and related conditions, which may be the main reason for deteriorating health among older people. A few research studies have concluded that retirement has little influence on health (Coe et al., 2012; Gorry et al., 2018).

There are no identified institutional or cultural differences between countries that support these different research findings. For example, Zhang’s findings in China differ from those in developed countries (Zhang et al., 2018), making it difficult to generalize findings from developed to developing countries. For this study, we selected China as representative of emerging economy nations because China has a sound system of compulsory retirement and well-established survey databases, including China Family Panel Studies (CFPS) and the China Health and Retirement Longitudinal Study (CHARLS). Hence, studying the situation in China will facilitate analysis of the health of retired people in other developing countries. We found that even in the context of China, scholars reach different conclusions. Some scholars argue that retirement has a negative impact on health. Lei was the first to find a significant negative impact on men’s health but not on women’s health (Lei et al., 2010). Zhang argued that retirees have more time to spend on health care and are therefore more likely to be diagnosed with health problems, leading to a deterioration in health status (Zhang et al., 2018). However, Che argued that retirement has a positive impact on the health status of white-collar men, suggesting that retirement may increase health-related exercise and the development of a healthier lifestyle, which in turn may have a positive impact on wellbeing (Bloemen et al., 2017; Che and Li, 2018). Despite conflicting interpretations, most empirical studies provide a theoretical framework for the concept that retirement affects health, although research on the mechanisms of effect is scant.

In summary, there are two main mechanisms through which retirement affects health. On the one hand, changes in health habits and social activities as a result of retirement become key avenues for influencing health. Retirement, on the other hand, produces changes in cognitive function in retirees, which has an adverse impact on their health.

As a result, this study will seek to address the following three questions that have emerged from previous research: (1) What impact does China’s existing retirement policy have on its citizens’ health? This study looks at not just how retirement affects Chinese retirees’ objective health, but also how it affects their subjective health. (2) If retirement has a significant impact on many types of health, what are the underlying mechanisms of action? Are they connected to lifestyle? (3) What role do key personal characteristics such as education level and marital status have on the health of retirees? Finding empirical answers to these questions will help clarify the causal relationship between retirement policies and residents’ health and the mechanisms underlying this relationship. Further, it will point to ways to improve health outcomes in the context of aging so as to fully acknowledge the value of elderly resources and the positive effect of “health in old age.”

Using data from CHARLS, we evaluated the self-assessed health and the objective health of older people, including depression and self-care ability, and employed an FRD model to assess the effects of retirement on different aspects of wellbeing. In addition, we studied the influence of retirement and personal traits on post-retirement lifestyle habits, elaborating on the ways retirement affects and/or changes health habits due to the influence of such factors as education and marriage. Chinese policies on compulsory retirement are mainly applicable to government agencies, enterprises, and public institutions, and males’ retirement ages are relatively concentrated. For the above reasons, this paper selected male respondents from government agencies, enterprises, and public institutions.

This study contributes to the field in several important ways. First, most previous researchers have looked generally at the overall impact of retirement on health, without classifying health into different types or making a comparative analysis of the various types. This is a primary reason for the controversy over the positive/negative effects of retirement on health. However, in this study, we looked at a wide range of indices measuring health and separated subjective health self-evaluation indices from objective health indices. Second, we analyzed the effects of retirement on behavioral and lifestyle factors that relate to health. Building on this, we further analyzed the mechanisms and paths through which retirement influences health. Third, there are inherent problems in the methods used in existing studies. For instance, retirement is not exogenous to health (Charles, 2002; Dave et al., 2006; Wang et al., 2011; Bonsang et al., 2012) and many endogenous factors have not been taken into consideration in the research on health. The FRD model was adopted in this study so that problems of reverse causality and endogeneity in the previous studies were avoided.

## Theoretical Background

### Compulsory Retirement System

The current Chinese policies on retirement have been in existence for a long time, with the *Labor Insurance Regulations of the People’s Republic of China* having established a basic framework for retirement ages in China (Giles et al., 2015; Feng et al., 2020). Subsequently, the *State Council’s Provisional Measures on the Resettlement of Older People, Weak, Sick and Disabled Officers*, and the *State Council’s Provisional Measures on the Retirement and Resignation of Workers* further standardized the country’s retirement policies. Table 1 gives an overview of the retirement ages for different genders and professions under various circumstances in China. The compulsory retirement age for most male individuals, apart from a small group of people including senior experts and high-ranking officers in government agencies, research institutions and public service agencies, is 60. For females, however, the regulations are more complex, with retirement age varying according to rank.

**TABLE 1:** Summary of Chinese policies on retirement age.

	Category	Male	Female
Under normal circumstances	Officer	60 years	55 years
	Worker	60 years	50 years
Under special circumstances	Complete loss of ability to work	50 years	45 years
	Disabled because of duty	Undefined	Undefined
	Special professions	55 years	45 years

These compulsory retirement policies are mainly applicable to government agencies, enterprises, and public institutions. Farmers and self-employed individuals, however, can retire as they need to, and so they are not included in this study. Furthermore, there is less variation in the retirement ages for men than for women. For these reasons, we selected male respondents from government agencies, enterprises, and public institutions. Such a sampling method facilitates the quasi-natural experiment of this study using the policies on compulsory retirement and the regression discontinuity-based recognition of the causal relationship between retirement and health.

### Subjective and Objective Health

Many previous studies have assumed that a self-evaluation of an individual’s health taken together with objective research serves as an appropriate and effective index for the assessment of overall health (Jylhä, 2009; Dowd and Zajacova, 2010). Self-assessments of health have been used in studies of older people in numerous countries (Stephan et al., 2010; Shrira et al., 2015), as they may augment objective health data with additional dimensions of physical, psychological and mental health and reveal details about medicines, disease control methods, and physiological functions (Kaplan and Baron-Epel, 2003). Ryan and Deci (2017) found that such subjective health evaluations had a positive correlation with intrinsic motivation (i.e., people were more likely to make choices according to their interests or needs).

A key finding of this study is that the reason the empirical results of previous studies have diverged so much (in addition to using different data sets) was most likely because they did not distinguish different types of health assessments and based their results on only self-evaluation of health (subjective health) or only objective indicators (objective health) in determining overall health. Yet, it is critical to distinguish between subjective health self-assessment markers and objective health indicators.

### Relationship Between Retirement and Health

It is generally perceived that retirement is good for health, since, from the perspective of objective health, retirees usually do not have heavy physical and/or mental workloads (Mazzonna and Peracchi, 2017), are able to enjoy more leisure time and opportunities to exercise, and experience less pressure (Latif, 2013; Kämpfen and Maurer, 2016; Jones et al., 2018). Given these benefits, increasing the retirement age would not reduce pressure on social security systems or increase labor supply. Rather, it would result in a significant increase in medical care expense and create additional social problems.

Some researchers, however, believe that retirement may lead to a deterioration in health, as the reduction in income which usually accompanies retirement may compel retirees to curtail health-related expenditures. At the same time, freedom from the constraints of working life may lead to a rise in unhealthy habits, such as excessive smoking, alcohol consumption and obesity, as has been seen among retirees in other countries (Chung et al., 2009; Behncke, 2012). In addition, separation from former colleagues may weaken retirees’ social networks, resulting in feelings of loneliness, alienation and cognitive decline (Mazzonna and Peracchi, 2017). From this perspective, it would seem feasible to alleviate labor shortage problems and current rising medical care expenditures by raising retirement ages (Behncke, 2012).

## Research Methodology

Endogeneity is a major research issue when it comes to the impact of retirement on health, and this has often been overlooked in past studies. Endogeneity may arise due to the omission of explanatory variables (there are many factors influencing health including some personal traits that cannot be observed), measurement deviations (such as continuing to work in retirement or stepping down from one’s duties ahead of official retirement), and reverse causality (individuals with poor health are more likely to retire) (Belgrave et al., 1987; Dwyer and Mitchell, 1999). Due to endogeneity, simply comparing health situations before and after retirement does not adequately reflect the causal effects of retirement on health. To address this problem, we adopted a regression discontinuity (RD) framework for the identification of causal relationships. RD is a quasi-random testing method. In cases where a random test is not possible, RD can effectively prevent the problem of endogeneity of parametric regression and provide a more accurate reflection of the actual causal relationship among variables (Lee and Lemieux, 2010).

Suppose that *y_i* is the variable of health output, which is one focus of this paper, *x_i*is the grouping variable, and *D_i* is the processing variable. Then, the health output variable of an individual affected by the retirement policy will be *y*_*1i*_. The variable that has not been processed will be *y*_*0i*_; the impact of policy will be *y*_1*i*_−*y*_0*i*_; the average processing effect will be E(*y*_1*i*_−*y*_0*i*_). The local average treatment effect (LATE) near discontinuity can be expressed as the following equation:


(1) $$LATE=E\left(y_{1\,i}-y_{o\,i}|x=c\right)=E\left(y_{1\,i}|x=c\right)-E\left(y_{0\,i}|x=c\right)=\lim_{x↓c}E\left(y_{1\,i}|x\right)-\lim_{x↑c}E\left(y_{1\,i}|x\right)$$

Regression discontinuity can be further divided into sharp regression discontinuity (SRD) and fuzzy regression discontinuity (FRD). SRD only occurs when the probability of processing an individual case rises from 0 to 1 at the point of discontinuity, while the FRD reflects the probability of processing an individual case that falls within (a, b), with 0 < a < b < 1.

Despite the existing Chinese system, not all the male respondents retire at age 60. For a small minority, such as essential skilled workers and senior professors, retirement may be delayed until age 65 (see Part II), while others may leave their positions before the official retirement age (a system known as “internal retirement”) because of health problems or other factors. Therefore, the probability of processing an individual case will not climb directly from 0 to 1 around the lawful retirement age of 60. Taking these points into consideration, this paper adopted FRD for the research analysis. For FRD, the two-stage least square (2SLS) is completely equivalent to the non-parametric estimation (Hahn et al., 2001). Therefore, only the regression results of 2SLS are presented in the empirical analysis.

Meanwhile, RD can be seen as a local random test, so whether or not covariates are added does not affect the consistency of discontinuity regression estimation. For that reason, this paper only includes the effects of processing variables on outcome variables in the benchmark regression. Covariates will be added in follow-up studies to observe whether they influence the health and living habits of the older people and to detect if there are any significant differences in the regression results.

## Data and Variables

### Data Sources

The data for this study was sourced from the China Health and Retirement Longitudinal Study (CHARLS). Similar to the EU’s SHARE, the United States’s HRS, the United Kingdom’s ELSA, and Japan’s JSTAR, CHARLS includes primarily individuals over age 45 and households whose members are in this age group. The questionnaire covers demographic information, household specifics, health conditions, medical care and insurance, career and retirement, pension, and respondents’ income and expenditures, and thus it offers high-quality micro data for interdisciplinary studies. The CHARLS national baseline survey was carried out in 2011 and included approximately 17,000 people from 10,000 households who were employed by or retired from 150 county-level and 450 rural institutions. In 2013, CHARLS followed up the baseline survey with a second national tracking survey which involved 18,000 individuals. The research in this paper is based on the combined dataset of the 2011 and 2013 surveys.

### Variables

#### Retirement Status and Age

The retirement variable is a dummy variable, such that if a respondent answers yes to the question “Have you retired (including early retirement) or stepped down from your duties (‘internal retirement’)?^1^” the retirement variable is 1; if the answer is no, it is 0. After analyzing the institutional background of the policy on compulsory retirement, we excluded farmers, self-employed individuals and households, and female respondents, thus limiting the sample to male respondents from government agencies, enterprises, and public institutions.

Additionally, the age variable of the respondents was extracted, and a dummy variable was set according to the legal retirement age of 60, such that if a respondent is over 60, the age variable is 1. Only respondents aged 50–70 were included in this study. The final sample size was 2,266.

#### Health Condition

In this paper, the Self-assessed Health (SAH) index, which is widely used internationally, was employed to measure subjective health. In the CHARLS questionnaire, different groups of respondents were asked to assess their health in the beginning and at the end of the health section respectively. The two groups were given different sets of response options. One included “Extremely good,” “Very good,” “Good,” “Average,” and “Poor”; the other, “Very good,” “Good,” “Average,” “Poor” and “Very poor.” In this paper, the best self-assessed health was valued as 1, while the poorest one was valued as 5. Therefore, the assessment score of each question ranged from 1 to 5.

In terms of objective mental health, the CHARLS questionnaire adopted the CES-D-10 scale^2^ to measure depression in respondents. The scores each respondent obtained from the 10 items^3^ in the scale were aggregated to measure depression, and there were four response options for each of these 10 items. In this paper, the score of the option indicating the highest degree of depression was defined as 3, and that of the option indicating the lowest degree of depression was defined as 0. The sum of each respondent’s scores from these 10 items was used to measure their mental health in terms of depression. The score of the depression index ranged from 0 to 30. A higher depression index indicated a lower level of mental health in a respondent.

To measure objective physical health, the Activities of Daily Living (ADL) index was employed. Respondents were given five daily activities (getting dressed, bathing, eating, getting out of bed, and using the bathroom) and asked to choose one of these four options for each activity: “It’s not difficult,” “It’s difficult but I can manage it,” “It’s difficult and I need help,” and “I can’t manage it.” The practice of Behncke (2012) was adopted in this paper: “It’s not difficult” was scored as 0, while the other three responses were each scored as 1. The scores each respondent obtained from the five activities were aggregated to get an ADL index which revealed their self-care ability in daily life. The ADL index ranged from 0 to 5.

All the physical, mental, and objective health indices are inverse ones, which means that a higher index indicates poorer health in the respondent.

#### Lifestyle Factors

CHARLS provides abundant data about the living habits of respondents, and in this study we focused on smoking, alcohol consumption, and sleeping habits. Smoking was defined as a dummy variable. If a respondent gave a positive answer (i.e., he still smokes) to the question “Do you smoke or have you quit smoking?” the smoking variable was valued as 1; otherwise, it was 0. Likewise, alcohol consumption was also defined as a dummy variable. If a respondent drank more than once per month, the alcohol variable was valued as 1; otherwise, it was 0. The sleep variable recorded the respondent’s average sleep time per night.

#### Personal Characteristic Variables

Our research considers the effects of two critical individual factors on respondents’ health condition and lifestyle: educational background and marital status. In our model, the educational level of each respondent and spouse was converted to the number of years of education. For example, a bachelor’s degree equated to 16 years. Marital status was 1 if the respondent was married; otherwise 0.

#### Descriptive Statistics

Table 2 shows the descriptive statistics of the important variables. Retired respondents accounted for 62% of our sample. The average age of respondents was 60.33. The Self-Assessed Health (SAH) average was 3.10, indicating that most respondents believed that their health was average. The Activities of Daily Living (ADL) average was 0.63, indicating that more than half of the respondents were able to manage the daily activities of getting dressed, bathing, eating, getting in and out of bed, and using the bathroom. The average depression score was 14.70, which was in the normal range. Responses to questions about lifestyle behaviors that affect health, such as sleep time, smoking and drinking alcohol, show that the average sleep time was 6.4 h, 48% of the respondents smoked, and 67% of them drank alcohol more than once a month.

**TABLE 2:** Descriptive statistics of the variables.

Variable	Sign	Observed value	Average value	Standard deviation
Retirement	Retirement	2,266	0.62	0.49
Age	Age	2,269	60.33	5.50
Self-assessed health	SAH	2,269	3.10	0.98
Activities of daily living	ADL	2,268	0.63	0.81
Depression	Depression	2,265	14.70	5.59
Sleep time	Sleep	2,180	6.40	1.51
Alcohol consumption	Alcohol	2,266	0.48	0.50
Smoking	Smoking	1,188	0.67	0.47

## Empirical Study and Analysis

### Discontinuity Effects of the Retirement Policy

**FIGURE 1:**
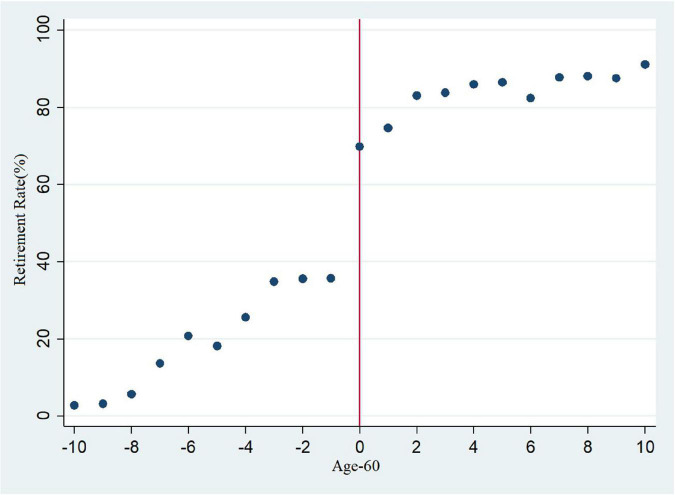
Age and retirement rate.

We used a scatter diagram to depict a qualitative analysis of the discontinuity effects of the system of compulsory retirement. If this system did not exist, the retirement rate should show steady changes as age increases. If discontinuity was detected around the ages specified by the system, it would be assumed that the difference in retirement was caused by exogenous factors (Dave et al., 2006; Bound and Waidmann, 2007). Figure 1 reveals the relationship between age and retirement and shows that the proportion of male respondents who retired at the age of 50 was less than 5%. The retirement rate gradually rose as age increased and reached 40% when respondents were approaching their 60th year. At the age of 60, the rate showed an obvious jump, hitting 70%. Thereafter, the rate continued to increase with age, and at age 70 the rate was above 90%.

As shown in Figure 1, there were notable discontinuities in the retirement rate before and after the age of compulsory retirement, and it cannot be totally ruled out that these discontinuities were caused by other noise. In the next stage, therefore, this study employed 2SLS for FRD.

The first stage of the 2SLS focuses on the effects of the retirement policy on the retirement rate (Garz, 2018). In this stage, the dependent variable of the regression is retirement, and the independent variable is the dummy variable of age and its polynomial. This stage covers the entire age range in our dataset. The regression results are shown in Table 3. In the first-stage regression, we controlled the fixed effects of province and time. The regression show that F statistic was higher than 10, indicating that the retirement policy had a significant positive effect on the retirement rate. This means that the dummy variable of legal retirement age was an effective instrumental variable. Specifically, columns (1) to (4) controlled, respectively, the first-, second-, third- and fourth-stage polynomial of the dummy variable, age.

**TABLE 3:** The first-stage regression: effects of the retirement policy on the retirement rate.

	(1)	(2)	(3)	(4)
Dependent Variable: Retirement				
Dummy variable: age	0.404*** (0.036)	0.337*** (0.057)	0.329*** (0.080)	0.311*** (0.103)
Constant term	0.469*** (0.029)	0.481*** (0.047)	0.434*** (0.065)	0.412*** (0.085)
Fixed effects of province	Yes	Yes	Yes	Yes
Fixed effects of time	Yes	Yes	Yes	Yes
Order of the Polynomial	1	2	3	4
R square	0.528	0.530	0.532	0.533
F statistic	341.6	167.8	254.5	55.71
Observed value	2,266	2,266	2,266	2,266

(1) The numbers in the brackets are robust standard deviations; (2) “*”, “**” and “***” indicate significant on the levels of 10, 5, and 1% respectively.

### Retirement and Health

The relationships between retirement and the health variables were obtained in the second stage of 2SLS (Grøtting and Lillebø, 2020; Picchio and van Ours, 2020). The regression results are shown in Table 4.

**TABLE 4:** The second-stage regression: effects of retirement on health and lifestyle habits.

Dependent variable	(1)	(2)	(3)	(4)	(5)	(6)
	SAH	ADL	Depression	Sleep	Alcohol	Smoking
Retirement	0.426** (0.196)	0.088 (0.144)	1.253 (1.083)	−0.733** (0.300)	0.074 (0.096)	−0.059 (0.134)
Constant term	2.852*** (0.124)	0.526*** (0.094)	13.957*** (0.757)	6.899*** (0.206)	0.420*** (0.068)	0.719*** (0.087)
Effects of year	Yes	Yes	Yes	Yes	Yes	Yes
Effects of province	Yes	Yes	Yes	Yes	Yes	Yes
Polynomial	Yes	Yes	Yes	Yes	Yes	Yes
Observed value	2,266	2,265	2,262	2,177	2,263	1,185

(1) The numbers in the brackets are robust standard deviations; (2) “*”, “**” and “***” indicate significant on the levels of 10%, 5% and 1% respectively.

According to our findings, retirement led to a higher SAH score at a significance level of 5%. In other words, retirement had a negative effect on subjective health. As for ADL and depression, retirement resulted in a slight increase in the probability of self-care inability and depression, but the effects were insignificant. This means that retirement did not cause any abrupt changes to physiological functions and health related to self-care ability. Meanwhile, retirement did not noticeably affect the respondents’ perception of depression. This may be due to the fact that most of the respondents were born in the 1950s or 1960s and had experienced dramatic changes to society and living conditions in their lifetimes, so retirement did not result in any sudden increase in depression among them.

Given the conclusions about self-assessed health and mental and physical health, we posit that for older people in China, the deterioration in subjective health after retirement may be attributed to unfulfilled intrinsic motivation. For instance, retirees may be compelled to accept a relatively lonely environment and lack opportunities to participate in activities that interest them. The worsening of subjective health is not caused by actual negative impacts on physical or mental health, such as disease or poverty.

**FIGURE 2:**
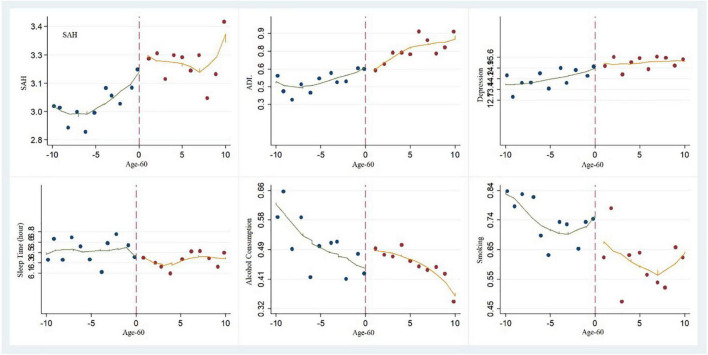
Local polynomial regression diagram: the breakpoint characteristics of health and living habits.

Figure 2 shows the smooth regression lines obtained from the process wherein the local linear function was adopted to apply the local polynomial to both sides of the discontinuity. The horizontal coordinate is “Age minus 60.” The vertical coordinates on the first line represent SAH, ADL and depression, respectively. It can be seen from the diagram that SAH showed a noticeable jump after age 60. This also verifies the regression results in Table 4.

### Retirement and Lifestyle Habits

As shown in columns (4) to (6) in Table 4, there was some difference in the effects of retirement on lifestyle habits. Retirement had a negative impact on sleep time. Compared to working individuals, retirees’ sleep time was reduced by 0.7 h per night. Based on the research results of the relationship between retirement and subjective health, we suggest that less sleep is not an intrinsically motivated choice on the part of older people but may be a result of sub-health problems like insomnia, which indirectly explains the reason for lower SAH scores. The impacts of retirement on smoking and drinking alcohol were not noticeable. The vertical coordinates on the second line of Figure 2 are sleep time, alcohol consumption and smoking, respectively. It can be observed from the graph that sleep time showed a marked drop after age 60. This also verifies the regression results in Table 4.

### Effects of Educational Background and Marital Status

Lee and Lemieux (2010) believed that adding covariates into the regression would not necessarily have any impact on the regression results (Imbens and Kalyanaraman, 2012), so we continued to add covariates to explore the possible effect of individual factors and thus ensure that our research was comprehensive. Educational background and marital status were taken as covariates in the empirical study, and the regression results are shown in Table 5. We found that a higher educational level was associated with a significant improvement in SAH, depression, and ADL, and higher education is associated with non-smoking. The influence of marital status on health was more limited. Being married had a significant effect on depression, and it is associated with a reduced tendency to smoke. However, neither a higher educational level nor having a spouse had much effect on drinking habits or sleep quality among our sample.

**TABLE 5:** Effects of educational background and marital status.

	(1)	(2)	(3)	(4)	(5)	(6)
	SAH	ADL	Depression	Sleep	Alcohol	Smoking
Retirement	0.413** (0.194)	0.048 (0.146)	1.055 (1.058)	−0.740** (0.301)	0.07 (0.097)	−0.051 (0.134)
Educational background	−0.034*** (0.012)	−0.053*** (0.010)	−0.319*** (0.072)	0.012 (0.020)	0.001 (0.006)	−0.015* (0.008)
Marital status	0.064 (0.104)	−0.04 (0.067)	1.895*** (0.576)	−0.287 (0.193)	−0.056 (0.051)	0.104* (0.059)
Constant term	3.034*** (0.141)	0.822*** (0.116)	15.610*** (0.842)	6.855*** (0.234)	0.421*** (0.078)	0.783*** (0.098)
Effects of year	Yes	Yes	Yes	Yes	Yes	Yes
Effects of province	Yes	Yes	Yes	Yes	Yes	Yes
Polynomial	Yes	Yes	Yes	Yes	Yes	Yes
Observed value	2,265	2,264	2,261	2,177	2,262	1,185

(1) The numbers in the brackets are robust standard deviations; (2) “*”, “**” and “***” indicate significant on the levels of 10%, 5% and 1% respectively.

Moreover, after the covariates were added into the initial regression, retirement still had significant impact on SAH and sleep time, signifying that the research outlined above was scientific.

### Robustness Test

We endeavored to estimate the effects of retirement on health (SAH, ADL and depression) and health-influencing factors (sleep time, alcohol consumption, and smoking) in the context of different bandwidths (Arai and Ichimura, 2016). From columns (1) to (5) in Table 6, the age intervals become progressively narrower. In column (1) the age range is 51–69, while in column (5) it is 55–65, which is the optimal bandwidth obtained by the cross validation methods (Clark and Royer, 2013). The study findings show that the parameter coefficient value declined with a narrower interval, while the impact of retirement on SAH was always significant, confirming that the estimated results of this paper were robust (Imbens and Kalyanaraman, 2012). In other words, retirement was associated with a lower SAH score and shorter sleep time among the respondents.

**TABLE 6:** Estimated results of different bandwidths.

	(1)	(2)	(3)	(4)	(5)
Choice of bandwidth	[51, 69]	[52, 68]	[53, 67]	[54, 66]	[55, 65]
SAH	0.566*** (0.209)	0.501** (0.232)	0.482** (0.245)	0.568** (0.272)	0.543* (0.307)
ADL	0.069 (0.149)	0.003 (0.166)	−0.091 (0.179)	0.081 (0.190)	−0.007 (0.230)
Depression	1.074 (1.172)	1.237 (1.314)	1.435 (1.353)	1.181 (1.491)	1.195 (1.782)
Sleep	−0.815** (0.325)	−1.067*** (0.371)	−0.975*** (0.374)	−1.006** (0.415)	−0.856* (0.479)
Alcohol	0.048 (0.107)	−0.013 (0.120)	−0.037 (0.121)	−0.159 (0.138)	−0.182 (0.162)
Smoking	−0.004 (0.145)	0.003 (0.156)	0.042 (0.161)	−0.036 (0.176)	0.005 (0.208)

(1) The numbers in the brackets are robust standard deviations; (2) “*”, “**” and “***” indicate significant on the levels of 10%, 5% and 1% respectively; (3) Cluster controlled the province-year-individual level; (4) This paper adopted the Akaike Information Criterion (AIC) to define the polynomial regression order.

We conducted robustness testing to assess the following issues:

(1) We tested the continuity of the conditional density function of the covariate at the point of discontinuity to ensure the feasibility of RD. The covariate was taken as a dependent variable for the regression of the equation in Table 4. The results show that there was not a significant rise at the point of discontinuity for the two covariates, educational background and marital status. This met the set requirements of RD (Thistlethwaite and Campbell, 1960; Bertanha and Imbens, 2020).
(2) We conducted a robustness test with a non-parametric approach (Jia, 2020), and the result was coincident with the 2SLS estimates.
(3) We adopted the method of McCrary (2008) to test whether there was evidence for bunching on one side of the discontinuity.

(2) $${\text{H}}_{0}:\theta \equiv \text{ln}\lim_{\text{x}↓\text{c}}\text{f}\,\left(\text{x}\right)-\ln\,\lim_{\text{x}↑\text{c}}\text{f}\,\left(\text{x}\right)\equiv {\text{lnf}}^{+}-{\text{lnf}}^{-}=0$$

According to the results of the empirical study, the estimated value of θ was 0.21 (its corresponding standard deviation was 0.12). On a significance level of 5%, the original assumption was that there was no evidence of bunching on one side of the discontinuity, which might indicate no manipulation of the assigned variable.

## Conclusion and Implications

We adopted the national baseline survey data from CHARLS, considered the Chinese policy on compulsory retirement, and used FRD to conduct an empirical study of the effects of retirement on the health and lifestyles of respondents (retired male individuals). Because China’s compulsory retirement policy is mainly applicable to state agencies, enterprises and institutions and males’ retirement ages are relatively concentrated while females’ individual position retirement ages are quite variable, the retirement age discussion is complicated. Consequently, we chose to focus only on government departments, enterprises and institutions and male respondents. According to our findings, retirement did not have a significant impact on objective health indices, including mental health (depression) and self-care ability (ADL). This seems to indicate that retirement has no obvious positive influence on objective health. This may be due to the fact that most respondents were born in the 1950s or 1960s and had experienced dramatic changes to society and living conditions in their lifetimes, so retirement did not result in any sudden increase in depression among them. This contrasts with Zhang’s findings (Zhang et al., 2018), possibly due to the various health metrics used. In Zhang’s study, outpatient incidents, physician visits, and hospitalizations were employed as objective health indicators, while in this study, physical health (ADL) and mental health (depression) were used. However, retirement had a pronounced negative impact on retirees’ perception of their own health, as measured by the SAH subjective health index. This is consistent with Lei’s study (Lei et al., 2010) and may relate to respondents’ unfulfilled intrinsic motivations. Such motivations could include: wishing to participate in social activities but not finding any suitable platforms to do so or insomnia shortening their sleep time. The negative effects, however, did not appear to be caused by extrinsic motivations or objective factors such as reduced income and/or changes to work schedule. Hence, we believe that it is feasible to formulate policies to bring retirees back to work or raise the retirement age, although such policies should leave room for individual choice. On one hand, this would satisfy older people’s needs for social interaction and make their lives more fulfilling, thus effectively addressing the problem of worsening SAH in retirees. At the same time, it could alleviate the problem of inadequate labor resources due to China’s aging population.

Second, the effect of retirement on lifestyle varied. Retirement shortened sleep times for older people, but its impact on smoking and drinking alcohol was not significant. Our findings regarding sleep times are consistent with the study by Ye (2018) and suggest that the poor sleep quality in retirement is a non-volitional behavior rather than an active choice on the part of the older people to sleep irregular hours. One possible explanation is that insomnia is caused by a change in the pace of life after retirement. Moreover, the decline in sleep quality was the main reason underlying the negative impact of retirement on subjective health. A study by Eibich (2015) concluded that retirees increase their sleep hours on weekdays, which may improve their level of self-reported well-being. This disparity with our findings may be traced to cultural traits. In underdeveloped nations, a lack of understanding of how to maintain one’s health and sleep properly may result in difficulty adapting to the changing pace of life after retirement. Our findings that the effect of retirement on smoking and drinking alcohol was not significant was in partial agreement with Ye (2018), who determined that retirement significantly reduced the likelihood of drinking and had no effect on smoking. In Ye’s study (Ye, 2018), middle-aged and older people maintained their pre-retirement lifestyles with respect to smoking and drinking after retirement, so there were no significant changes to their objective health, mentally or physically.

On one hand, this is positive in preventing the sudden decline of objective health due to retirement, but on the other, it indicates that the reduction in stress post-retirement failed to motivate respondents to adopt a healthier way of life. Therefore, it is suggested that commercial and non-profit psychological consultation services, social platforms and recreational centers for older people be further supported in order to provide this demographic with appropriate mental support and more options in the way of social activities. Efforts should be made to improve the health of older people by satisfying their intrinsic motivations and needs rather than simply increasing investment in senior medical care. This would not only benefit older retirees and those who have postponed their retirement but would also make better use of medical resources in the post-epidemic era.

Third, when education and marital status were added into our model, we noted that a higher level of education improved SAH as well as objective physical and mental health. These also deterred middle-aged and older people from smoking. This is consistent with the Che and Lei studies (Lei et al., 2010; Che and Li, 2018), which demonstrated that highly educated people have more health-related knowledge and are more likely to maintain healthy lifestyles after retirement. Marital status merely influenced the mental health of respondents and reduced the tendency to smoke. However, neither educational level nor marriage was seen to alleviate the problems of alcohol consumption and poor sleep among older people. This is in keeping with Chinese society today, as public education and spousal advice often tend to focus on the harmful effects of smoking while paying little attention to alcohol consumption and insomnia, in the absence of scientific intervention. Hence, our results suggest that communities should redouble efforts to promote healthy living and offer relevant assistance to older people to improve their living habits. As education and marriage do not have much direct impact on the problems of drinking and poor sleep among elders, emphasis should be placed on health guidance.

This paper makes some important contributions to the existing body of research on the issues concerned. First, this study incorporated a range of indices to measure health and separated subjective health self-evaluation indices from objective health indices. Our results show that retirement has differing impacts depending on the type of health index, and this can partially explain the controversy over the relationship between retirement and health that has emerged from previous studies. Second, our research focused on the effects of retirement on behaviors and lifestyles which are closely related to health. Building on this, we further analyzed the mechanisms and paths through which retirement influences health. Third, the FRD model was adopted in this study, so the problems of reverse causality and endogeneity in previous studies were avoided.

However, there are some limitations to our study. For instance, the samples included in this study were comprised of male individuals from government agencies, enterprises, and public institutions. Hence, the conclusions of this paper may not apply to all retired people in China (such as self-employed individuals and females). Also, due to the limitations of available data, it was not feasible to conduct a cross-period comparative study on the same sample. In future research, we plan to address these limitations by expanding our sample to include females and self-employed individuals and conduct a panel data study.

## Data Availability Statement

The original contributions presented in the study are included in the article/supplementary material, further inquiries can be directed to the corresponding author/s.

## Author Contributions

YT reviewed the literature, proposed the research model, and designed the study. DL conducted the literature search, proceeded with the data extraction process, and involved in the development of the manuscript. SM conducted the statistical analysis and revised the manuscript critically for important content. QW wrote the first draft of the manuscript. SG revised the manuscript and rechecked the relevant data of the manuscript. SI put forward many constructive suggestions on promoting the revision of the manuscript and supervised the entire writing process of the manuscript. All authors have approved the final manuscript to be published.

## Conflict of Interest

YT was employed by company Guangzhou Rural Commercial Bank. The remaining authors declare that the research was conducted in the absence of any commercial or financial relationships that could be construed as a potential conflict of interest.

## Publisher’s Note

All claims expressed in this article are solely those of the authors and do not necessarily represent those of their affiliated organizations, or those of the publisher, the editors and the reviewers. Any product that may be evaluated in this article, or claim that may be made by its manufacturer, is not guaranteed or endorsed by the publisher.

## Footnotes

1 “Retirement” here refers to retirement from a government agency, enterprise or public institution, excluding retirement for accessing rural old-age pension.

2 Radloff tested the reliability and validity of the scale and believed that the scale could effectively evaluate the depression of the respondents.

3 These 10 items are as follows: I was bothered by things that don’t usually bother me; I had trouble keeping my mind on what I was doing; I felt depressed; I felt that everything I did was an effort; I felt hopeful about the future; I felt fearful; my sleep was restless; I was happy; I felt lonely; I could not get “going.”
